# Digital competence for emergency remote teaching in higher education: understanding the present and anticipating the future

**DOI:** 10.1007/s11423-023-10194-4

**Published:** 2023-02-07

**Authors:** Henry Cook, Tiffani Apps, Karley Beckman, Sue Bennett

**Affiliations:** University of Wollongong, Wollongong, Australia

**Keywords:** Digital competency, Higher education, COVID-19, Teachers

## Abstract

Higher education has increasingly adopted online and blended models of teaching. Guided by institutional policy and digital competence frameworks, the integration of digital tools and competences is perceived as essential. The pivot to emergency remote teaching (ERT) in response to the COVID-19 pandemic increased the use of digital technologies and the need to deploy and support digital competences. Researchers captured a range of remote teaching practices in higher education across this period that highlight the adaptability of teachers despite a lack of preparation for such an event. This study reviewed empirical studies of ERT from the past 2 years to derive a conceptual frame for ERT digital competence, which was then applied as a lens to analyse teaching or digital competency frameworks from Australian universities. The findings of this paper demonstrate the pre-pandemic teaching and digital competency frameworks captured digital competencies relevant to ERT in varied ways. Practically, the findings provide a starting point for understanding digital competences needed for ERT to ensure future preparedness in responding to a crisis that disrupts educational provision. We also suggest universities can better support the development of teachers’ digital competence through practical operationalisations that connect technical and pedagogical knowledge, make digital possibilities across modes of delivery explicit, and acknowledge the need to protect wellbeing of educators.

## Introduction

Higher education has increasingly adopted online and blended models of teaching. This practice has been guided by institutional policy and strategy that position the integration of digital tools and competencies as essential to meet the needs of labour markets and remain relevant in a digital society (Webb et al., 2021). The digitisation of teaching and learning requires digitally competent teachers (Sharpe et al., 2022). The conceptualisation of digital competency, however, is contested, between being a tangible skill to develop or an ongoing practice to be supported (Zhao et al., 2021). Within this context, the COVID-19 pandemic resulted in a widescale pivot to emergency remote teaching (ERT), increasing the use of digital technologies and the need to deploy and support teacher digital competencies. Researchers have captured a range of emergency remote teaching practices in higher education across this period (Lin & Johnson, 2021). As higher education institutions move beyond the pandemic, it is not clear what digital practices will continue nor how institutions should support continue to support the ERT practices that have emerged.

The aim of this paper is to review empirical literature that reveals university teachers’ ERT digital competence and map that to existing operationalisations of digital competence expressed in university policy documents. Building on contributions of the special issue, *Shifting to digital* (Lin & Johnson, 2021), this paper also contributes to understanding the challenge of rapid shifts in digital practices and supports for teachers in higher education. The paper presents a document analysis of publicly available digital and teaching capability frameworks from 10 Australian universities. The analysis was guided by a conceptual frame developed from a synthesis of the empirical literature concerned with teachers’ digital competencies over the first 2 years of the COVID-19 pandemic. The analysis brings together pre-pandemic understandings of digital competencies in higher education with teachers’ bottom-up responses to the COVID-19 pandemic. This understanding is important for ensuring ERT is reflected digital competence frameworks so that teachers are prepared for future crises that disrupt education, whether they be global or local, and to consider how competence frameworks might best reflect the aspects of digital competence all higher education teachers need.

## Background

Digital competence is broadly defined as a set of skills required for participation in a specific context or society (Zhao et al., 2021). Digital competency in higher education has been a topic of debate, positioned between a list of defined skills that individuals possess through to more comprehensive definitions of socio-cultural digital practices (Spante et al., 2018). There are also variations in the numerous institutional, governmental, and societal frameworks which exist to describe digital competency across teacher profiles (eg. Crompton, 2017; JISC, 2019; Redecker, 2017). Within higher education, digital competence that teachers need for teaching and learning is operationalised within two types of institutional documents—teaching competency frameworks and digital competency frameworks. Within Australian higher education, the conceptualisation of teacher digital competencies has been informed by international models, such as JISC (Press et al., 2019). Most digital competency frameworks focus on technical or operational aspects of digital competence, with few addressing the effects on pedagogy and curriculum (Falloon, 2020). Thus, there is a need to better understand the ways that digital competence is enacted in various modes of digital education and how educators are supported to develop digital competence for teaching and learning.

Research has called for institutional leadership to guide innovation and understand the contextual factors in applying these frameworks and supporting the development of teacher digital competency (Pettersson, 2018). Similarly, attention needs to be paid to the pedagogical application of digital competence by teachers to understand the variety of innovative digital practices and to support teachers’ practice (Scherer et al., 2021; Zhao et al., 2021). In reconciling this complex set of considerations about digital competence, it is tempting for institutional standards frameworks to reduce capabilities to checklists (Falloon, 2020). In moving from reductive views of digital competency, digital competency is more than just technical proficiency, instead encompassing both use and understanding of the myriad of digital modalities and concerns which come with digital technologies in people’s lives (Janssen et al., 2013).

The digital competence of teachers in higher education is varied (Esteve-Mon et al., 2020; Zhao et al., 2021). Universities have made considerable investments in supporting teachers to become educators who can leverage different modalities and platforms that are assembled into modern learning environments. Supports are materialised in an array of different artefacts for teachers including professional networks, development programs, help resources and frameworks. Yet, there is still a need for research to explore how teachers develop digital competency (Tondeur et al., 2012).

### COVID-19 and digital competence—an opportunity to learn and reflect?

Prior to COVID-19, digital competency had long been considered a desirable skillset to participate within an exceedingly digital society and was often framed as a deficit in teachers whose proficiency ranged from novice to mastery (Selwyn, 2007). Research on teachers’ digital competence tended to focus on a duality between digital and pedagogical approaches, with teaching being a prioritised skillset for faculty and digital as emergent (Falloon, 2020).

With the rapid shift to ERT during the COVID-19 period, teacher digital competency became a necessity for all. Universities quickly increased the provision of digital services, and moved teaching, learning and support services to online modes (Webb et al., 2021). The speed and scale of this response placed significant demands on the teaching and digital competencies of all educators across education sectors. While some universities were better prepared than others, the sudden activation of digital education resulted in “just-in-time” approaches to teaching that leveraged and developed skillsets through local contextual supports and the broader higher education community (Crawford et al., 2020). Although continuity of education was achieved during this time, inequalities were exposed in both teachers’ and students’ access to digital technologies and their varying levels of digital competence (Webb et al., 2021). While researchers have sought to capture teaching and learning practice across the ERT period, there is a need to better understand how teachers enacted and developed their digital competency across this period, and how this may inform future practice.

## Methodology

The broad aim of this qualitative study was to review the empirical literature about university teachers’ digital competence from the ERT period and analyse existing operationalisations of digital competence in university contexts. This paper explores the following research questions:

- RQ1. How can university teachers’ ERT digital competence be characterised from the available empirical literature?
- RQ2. How do existing institutional competence frameworks align with university teachers’ ERT digital competence as characterised from the available empirical literature?

To do this, the study was conducted in two phases. In Phase 1, we undertook a review of empirical literature to derive a series of normative statements to create a conceptual frame characterising university teachers’ digital competence from the ERT period. In Phase 2 we analysed digital or teacher capability frameworks selected from 10 Australian universities using the Phase 1 normative statements as an analytic framework.

### Phase 1 characterising university teachers’ ERT digital competence

The purpose of this phase was to review the empirical literature about university teachers’ digital competence across the ERT response to the COVID-19 pandemic and develop a conceptual frame that captured these digital competencies. To develop the conceptual frame a literature search was conducted. The search was conducted across Web of Science, SCOPUS and Google Scholar using the following keywords and “digital”, “education”, “higher education”, “COVID-19” and “competence*”. The following inclusion criteria were applied to the results:

- Peer-reviewed journal articles published during the ERT period (from 2020 through to November 2022), and
- Empirical research findings on aspects of teacher digital competency.

Fifteen articles met the criteria above for inclusion in the literature review.

Thematic data analysis was conducted using the following four steps:

1. The research papers were first analysed inductively to identify and characterise the findings about teachers’ digital competence. This initial round of analysis developed a preliminary set of codes that represented aspects of teachers’ digital practices during ERT.
2. The second phase organised the codes into broad thematic categories.
3. The third phase developed the conceptual frame. During this phase the research team reviewed each thematic categories and associated codes to create normative statements of digital competence. The normative statements express the capabilities a teacher should develop to demonstrate an aspect of digital competency.
4. The normative statements, thematic categories and associated codes were then interrogated through the application of key questions to ensure quality, coherence and internal consistency (Braun & Clarke, 2013).

Eight normative statements were derived from the reviewed research literature which applied in Phase 2.

### Phase 2 analysis of university frameworks

Ten Australian university frameworks were selected for Phase 2 of the study. The selected frameworks included digital competence frameworks and teaching capability frameworks. The digital competence frameworks focused on the operationalisation of teachers’ digital competencies for teaching and learning, while the teaching capability frameworks focused more broadly on teaching and learning capabilities within which digital competencies were embedded. The frameworks were sampled from 36 public universities in Australia with publicly available frameworks. At the time of analysis, the teaching or digital capability frameworks of four universities were under review. A sample of 10 frameworks was selected for maximum variation in university type including geographic location (regional/city and across states), ranking and available delivery modes. The selected frameworks were from universities across 5 of the 8 Australian states and territories, and include 2 regional and 8 city universities, 2 highly ranked universities (top 100 QS World University ranking), and 5 universities with extensive online delivery offerings. Of the 10 selected frameworks, 3 are characterised as digital competence frameworks (DC1, DC2, DC3) and 7 as teacher capability frameworks (TC1–TC7). All 10 frameworks were in place prior to or at the beginning of the pandemic, thus capturing the conceptualisation and operationalisation of teachers’ digital competencies in Australian universities prior to the ERT period.

Phase 2 data analysis was deductive in nature. The research team applied the conceptual frame developed in Phase 1 to the ten selected university frameworks. The aim of this analysis was to compare the existing frameworks with the normative statements for alignment or additional understanding of digital competence in practice. An initial reading of each framework was conducted by the research team. Following this, the research team applied the conceptual frame by coding each framework for explicit reference to the normative statement and/or descriptor. The codes were reviewed for consensus across the research team.

## Findings

The findings are presented in two sections, addressing the research questions.

### 1. RQ1 How can university teachers’ ERT digital competence be characterised from the available empirical literature?

Fifteen empirical articles that examined teachers’ digital competence across the ERT period were included for review in this study. The 15 studies included 9 high-response surveys of teachers (ranging from 50 to 1000 respondents) drawing on interdisciplinary pools of teachers (Bartolic et al., 2021; Damşa et al., 2021; Kaqinari et al., 2022; Mishra et al., 2020; Myyry et al., 2022; Scherer et al., 2021; Shrestha et al., 2022; Väljataga et al., 2020; Watermeyer et al., 2021) and 6 descriptive case studies (Dalipi et al., 2022; Gao & Zhang, 2020; Moustakas & Robrade, 2022; Müller et al., 2021; Oliveira et al., 2021; Scull et al., 2020). Three major themes were constructed through an analysis of the findings presented in the literature reviewed: technologies, preparedness, and experience. The code descriptors and derived normative statements are presented in Table 1 and "Appendix" presents an overview of themes across the reviewed articles.

**Table 1:** Phase 1 themes and the derived normative statements of teachers’ ERT digital competence

Themes	Code descriptors (derived from literature review)	Normative statements of ERT digital competence (conceptual frame)
Technologies	Teachers’ use of technologies was focused on familiar tools	N1. Selects digital technologies that are appropriate for teaching and learning contexts
	Teachers’ selection of technologies was guided by university technology infrastructures	
	Teachers prioritised digital technologies which supported their connection with students	
	Teachers identified the technical limitations within their digital learning environments	N2. Accommodates technical challenges in teaching practice and learning designs
Preparedness	Teachers leveraged informal digital practices into digital learning environments	N3. Transfers competencies to adapt to a range of learning contexts and modes
	Teachers embraced emergency remote teaching rather than completely adopt an online delivery model	
	Teachers assembled and adapted digital learning environments to support their pedagogical intentions	N4. Understands pedagogical demands of discipline, context, and digital learning environments
Experience	Teachers relied on institutional and collegial networks to refine their digital learning environments	N5. Connects with networks of support and resources to refine practice
	Teachers supported students through a pastoral approach within digital learning environments	N6. Supports students to navigate the demands of digital environments through design
	Teachers considered the student experience when engaging with digital learning environments	
	Teachers sought to iteratively develop their digital learning environment through reflection and student evaluation of the learning experience	N7. Reflects on and evaluates the integration of digital technologies over time
	Teachers recognised the impact of emotion, increased workload and stretched goodwill on the design and delivery of digital learning environments	N8. Assesses the time demand and value of digital technology integration

The conceptual frame, comprising the eight normative statements of digital competence, provides a bottom-up characterisation of digital competence from across the ERT period. A brief outline of the review findings for each theme is presented below in connection with derived normative statements of teachers’ digital competence.

#### Technologies

The selection and use of technologies was a key component of teachers' ERT digital competence. Teachers selected technologies based on four factors: familiarity, availability, functionality, and technical competence. Teachers' selection of technologies for use during ERT was typically driven by familiarity with available technologies as several studies reported educators refrained from adopting new technologies to support learning and teaching (Bartolic et al., 2021; Damşa et al., 2021; Müller et al., 2021; Väljataga et al., 2020). Most teachers relied on their existing digital practices, such as uploading recordings and synchronous messaging, as these had been utilised as part of their day-to-day teaching prior to emergency delivery (Bartolic et al., 2021; Dalipi et al., 2022; Damşa et al., 2021; Kaqinari et al., 2022; Oliveira et al., 2021). While many also expressed limited experiences with completely online delivery, the connection between existing practices and these technologies helped inform pedagogy during this time (Gao & Zhang, 2020; Müller et al., 2021).

Teachers’ selection of technologies was primarily guided by the availability of university technology infrastructure. Several studies found that teachers felt the types of technologies and digital infrastructures provided through their respective universities were appropriate and this guided their use (Bartolic et al., 2021; Dalipi et al., 2022; Gao & Zhang, 2020; Väljataga et al., 2020; Watermeyer et al., 2021). This was most likely an outcome of the emergency context as teachers lacked the time to explore other technologies and the movement online was mandated by their institutions (Damşa et al., 2021; Moustakas & Robrade, 2022; Scherer et al., 2021). Teachers prioritised technologies based on functionality by selecting tools that supported communication and connection with their students. Communication technologies served an important role in emergency delivery as platforms through which teachers could conduct learning activities (Gao & Zhang, 2020; Mishra et al., 2020; Moustakas & Robrade, 2022; Shrestha et al., 2022) as well as facilitate classroom connections and management (Scull et al., 2020; Väljataga et al., 2020).

Teachers selected technologies based on their own perceived technical competence. This included being able to identify and provide technical support for students because the usual support services were disrupted or overwhelmed with increased demand. Examples included diagnosing problems with network connections (Bartolic et al., 2021; Mishra et al., 2020; Moustakas & Robrade, 2022; Shrestha et al., 2022), developing new technology solutions during delivery (Gao & Zhang, 2020; Oliveira et al., 2021) and finding new ways to develop content online (Väljataga et al., 2020).

Two normative statements of digital competency were derived from the research findings associated with teachers’ digital competence in the context of technologies used and associated challenges:

- N1. Selects digital technologies that are appropriate for teaching and learning contexts.
- N2. Accommodates technical challenges in teaching practice and learning designs.

#### Preparedness

Teachers' preparedness for ERT was a key factor associated with digital competency across the reviewed studies. Preparedness was varied, drew on informal digital practice, acknowledged the temporality and distinctness of ERT, and shaped the ways that teachers assembled digital learning environments.

Teachers leveraged their informal digital practices in their teaching. Teachers sourced information from their everyday social networks to creatively inform their teaching methodologies (Damşa et al., 2021). The use of everyday technology practices provided teachers an existing digital competency to leverage when adopting and adapting existing resources to online delivery (Väljataga et al., 2020). Teachers acknowledged the temporality and distinctness of ERT. Multiple studies found that the level of preparedness was not found to be a direct indicator of a simple transition to ERT (Bartolic et al., 2021; Kaqinari et al., 2022; Müller et al., 2021). Several studies found that teachers were explicitly approaching this delivery period as different from typical online delivery with little expectation that this type of delivery would be continue into the future (Bartolic et al., 2021; Dalipi et al., 2022; Müller et al, 2021; Watermeyer et al., 2021). Väljataga et al. (2020) found that although a high percentage of teachers saw the value in some of the changes they had made to their delivery during this period there was little expectation that the change would be adopted in their overall teaching approach. The ERT experience, whether teachers felt these practices would continue or not, still resulted in an increase in teacher competency and confidence with digital technologies (Myyry et al., 2022).

Teachers assembled digital learning environments to support their intended pedagogical approaches. The impact of the ERT on higher education was profound with stakeholders and systems unprepared for such a sudden shift, leaving teachers to rearrange pedagogical practices in less-than-ideal forms (Dalipi et al., 2022; Kaqinari et al., 2022; Moustakas & Robrade, 2022; Müller et al., 2021; Oliveira et al., 2021; Watermeyer et al., 2021). Primarily these findings support the view that teacher preparedness was developed through the contextual transformations of learning experiences and feedback from their students (Scherer et al., 2021; Scull et al., 2020; Shrestha et al., 2022).

Three normative statements of digital competency were derived from the research findings associated with the varied ways teachers leveraged their digital competence to respond to the demands of ERT:

- N3. Transfers competencies to adapt to a range of learning contexts and modes.
- N4. Understands pedagogical demands of discipline, context, and digital learning environments.

#### Experience

Teachers’ experiences of ERT highlighted key aspects of their digital competence including the ongoing role of formal support networks, the provision of student support, understanding the digital demands placed on students, understanding the demands on themselves, and the role of reflection and evaluation.

Teachers relied on formal support networks to develop their digital competence. Three studies detailed teachers feeling sole responsibility for preparing their learning environments because their confidence in supports provided was mixed (Damşa et al., 2021; Kaqinari et al., 2022; Väljataga et al., 2020). Other studies reported that teachers valued institutional supports in the pivot to ERT (Bartolic et al., 2021; Watermeyer et al., 2021). Institutional support was found to be most effective when collegial and direct communication was facilitated, especially when teacher confidence in online learning was low (Mishra et al., 2020; Moustakas & Robrade, 2022; Müller et al., 2021; Scherer et al., 2021). Complementary to institutional support online networks, shared practices across the academic community also provided a meaningful platform for teachers to connect and develop competency (Dalipi et al., 2022; Damşa et al., 2021; Oliveira et al., 2021; Shrestha et al., 2022).

Teachers were required to facilitate student support to cater to the diversity of students’ digital competence. Several studies found teachers during this emergency delivery identified their pastoral roles in online learning as being equally important as their technical skills when working with the diversity of learner needs (Scull et al., 2020; Watermeyer et al., 2021). This saw teachers having to enact digital skill sets that could enable them to connect with their cohorts, as well as develop their abilities to interpret and respond to student digital learning (Väljataga et al., 2020). Meanwhile, Moustakas & Robrade (2022) and Mishra et al. (2020) found teachers struggled to maintain student motivation because they felt less able to respond to students' moods or facial expressions online.

Teachers also considered the digital demands placed on students when designing digital learning environments for ERT. Digital learning environments were conceptualised through teachers understanding their students’ needs, integrating their existing in-person practices into online teaching (Gao & Zhang, 2020). Student engagement and accessibility were particularly difficult challenges both synchronously and asynchronously through digital learning environments (Dalipi et al., 2022; Müller et al., 2021; Scull et al., 2020). Bartolic et al. (2021) reported that many teachers felt they were still able to enact meaningful learning experiences and flexible assessment without compromising academic standards.

ERT placed significant demands on teachers. Teachers experienced limited time to develop their ERT, increased academic workloads and impacts of the unstructured nature of delivery (Dalipi et al., 2022; Oliveira et al., 2021; Shrestha et al., 2022; Väljataga et al., 2020; Watermeyer et al., 2021). Teachers detailed the significant overhead of constructing ERT environments that effectively leveraged the technologies and their contextual knowledges to support students’ engagement (Kaqinari et al., 2022; Moustakas & Robrade, 2022; Müller et al., 2021; Scherer et al., 2021).

Teachers engaged in reflection and evaluation to iteratively develop digital learning environments. The emergency response was seen by some as an opportunity to revisit content as well as reconsider how it could be taught (Dalipi et al., 2022; Kaqinari et al., 2022; Müller et al., 2021). Resources had to be reviewed and made more concise as students had to understand key concepts to ensure strong engagement (Scull et al., 2020). Digital interactive activities however saw little adoption because teachers had insufficient time to devote to redesigning digital learning environments (Damşa et al., 2021).

Four normative statements of digital competency were derived from the research findings associated with teachers’ experiences of ERT:

- N5. Connects with networks of support and resources to refine practice.
- N6. Supports students to navigate the demands of digital environments through design.
- N7. Reflects on and evaluates the integration of digital technologies over time.
- N8. Assesses the time demand and value of digital technology integration.

In sum, these eight normative statements of teachers' digital competence were derived from the review of the 15 selected research papers exploring teachers’ digital competence during ERT.

### 2. RQ2 How do existing institutional competence frameworks align with university teachers’ ERT digital competence as characterised from the available empirical literature?

The purpose of this second phase of the study was to apply the conceptual frame as an analytic tool to determine the extent to which teachers' ERT digital competence align with digital competence in practice across a sample of Australian universities. An overview of the alignment of the conceptual framework and the teaching and digital competency frameworks from 10 Australian universities is provided in Table 2 followed by a discussion of each normative statement.

**Table 2:** Alignment between normative statements and university frameworks

Normative statements of digital competence	Universities frameworks									
	TC1	TC2	TC3	TC4	TC5	TC6	TC7	DC1	DC2	DC3
N1. Selects digital technologies that are appropriate for teaching and learning contexts	X	X	X	X	X	X	X	X	X	X
N2. Accommodates technical challenges in teaching practice and learning designs								X	X	X
N3. Transfers digital competencies to adapt to a range of learning contexts and modes	X			X	X	X		X	X	X
N4. Understands pedagogical demands of discipline, context, and digital learning environments		X	X		X		X			
N5. Connects with networks of support and resources to refine digital practice	X	X	X	X	X			X		X
N6. Supports students to navigate the demands of digital environments through design		X	X	X	X		X	X	X	X
N7. Reflects on and evaluates the integration of digital technologies over time	X	X	X		X				X	X
N8. Assesses the time demand and value of digital technology integration								X	X	X

#### N1. Selects digital technologies that are appropriate for teaching and learning contexts

The selection of digital technologies in teaching and learning was present in all ten frameworks. Statements of competency within these frameworks focused on teachers’ selecting digital technologies suitable for specific pedagogies or student needs. These included facilitating or supporting student learning generally (DC1, DC2, TC7), facilitating student collaboration and engagement (TC1, TC3, TC4, TC5, TC6, DC3), facilitating flexible learning (across locations and time zones) (TC2, TC5), and enhancing course assessment and feedback (TC3, TC4).

The alignment between Normative Statement 1 and all ten university frameworks is not surprising given the foundational nature of this aspect of digital competence. One difference that was observed between the literature review findings on ERT digital competence and current frameworks was the scope of teachers’ access to digital technologies. The ERT literature review findings highlight the ways teachers relied on existing technologies procured or approved by the university. Yet, university frameworks did not specify the selection of a specific suite of technologies procured by the institution, but rather framed technology selection as a creative pursuit that make a teacher’s practice more “engaging, effective and efficient” (TC3). The distinction between these approaches suggests teacher design practices prior to the pandemic were more open. As outlined in Kanjanapongpaisal & Antee (2021), the selection and design of technology use within a face-to-face or blended course is usually driven by individual educators, while courses predominantly delivered online are likely to be co-designed or supported by academic development or learning design teams. This support was not typically available during the ERT period, and thus teachers' learning designs and choices were shaped by their own experiences, skills, and knowledge, leveraging what was provided to them to meet institutional requirements and student expectations.

#### N2. Accommodates technical challenges in teaching practice and learning designs

Accommodation of technical challenges was evident in three frameworks (DC1, DC2, DC3). All three were digital capability frameworks and included practices such as finding solutions or workarounds (DC2) and managing technical issues (DC1, DC3). Seven studies captured teachers’ frustration, as well as the resolve, that came from troubleshooting technical issues. When designing for learning teachers considered what was in their control including reconfiguring technologies, what was out of their control such as internet bandwidth, and where they negotiated control such as shared solutions with students (Bartolic et al., 2021; Dalipi et al., 2022; Gao & Zhang, 2020; Mishra et al., 2020; Oliveira et al., 2021; Shrestha et al., 2022; Väljataga et al., 2020).

Perhaps not a surprise, the teaching capability frameworks reviewed in this study did not explicitly include teachers’ competencies to accommodate technical challenges within their learning designs. The omission of technical competence suggests that technical aspects are viewed as distinct from teaching and the role of institutional technical support rather than university teachers’ work (Kebritchi et al., 2017). However, the literature review findings showed that teachers were needed to solve technical challenges themselves while teaching, and also needed to mitigate potential technical challenges through the considered design of their digital learning environments.

#### N3. Transfers digital competencies to adapt to a range of teaching contexts and modes

Seven frameworks detailed competencies that aligned with the notion of transferability of general skills and knowledge to adapt to varied teaching modes or contexts. Transferring digital competencies to adapt to a range of teaching contexts and modes was evidenced as a technical skill in one university framework (DC2). This digital capability framework highlighted the need for teachers to be able to “work across a range of devices and services (personal and institutional)”. Two frameworks referred to blended learning environments (DC1, DC3), while four focused on implementing innovation and adaptability of teaching practice, without specifying a digital focus (TC1, TC4, TC5, TC6).

While the literature we reviewed acknowledges the distinctive nature of ERT, the importance of transferring digital practices to adapt to a range of contexts continues to be a key digital competence. Yet, only one university framework explicitly referred to teachers’ transferring and adapting digital practice as a competence. The other six frameworks made more general references to the transfer of pedagogical skill and knowledge across a range of contexts. A more general description of skill transfer provides space for inclusion of a teachers’ pedagogical skills and knowledge including digital practice. However, a lack of detail or explicit mention of the digital in university frameworks can serve to obscure digital possibilities (Falloon, 2020), particularly for teachers with lower digital confidence and competence.

#### N4. Understands pedagogical demands of discipline, context and digital learning environments

Four frameworks contextualised pedagogical demands within teaching contexts. One referred to teachers’ capability to critically evaluate the role of technologies in the learning experience and ensure relevance (TC7). Three referred to being able to leverage discipline knowledge, approaches and resources in student learning (TC2, TC3, TC5). These statements contribute to an understanding of teachers as disciplinary experts who harness resources and contexts to effectively share knowledge with students. While the pedagogical demands of disciplines and contexts were present both in the ERT literature and the university frameworks reviewed in this study, the alignment with digital aspects of learning and environments was implied rather than explicitly stated.

Prior to ERT, teachers adapted learning designs in response to contextual requirements (Bennett et al., 2017). The importance of the alignment between discipline demands, learning objectives, digital resources and, more broadly, digital learning environments was made more evident during ERT. The pedagogical approaches captured in the ERT literature illustrated the ways that teachers were unprepared to bring together their discipline knowledge with the digital demands of online learning environments (Müller et al., 2021; Myyry et al., 2022; Oliveira et al., 2021; Watermeyer et al., 2021). Yet teachers developed competency in assembling digital tools and experiences to meet the pedagogical demands of their discipline over time. This digital competency was shaped by student feedback and teacher reflection (Bartolic et al., 2021; Gao & Zhang, 2020; Müller et al., 2021; Oliveira et al., 2021).

#### N5. Connects with networks of support and resources to refine digital practice

Connecting with networks of support and resources to refine digital practice was evidenced in two frameworks (DC1, DC3). One builds on JISC’s (2019) digital competency model, referring to networks of support as a component of information literacy for critically engaging with digital information (DC1). The other positions networks of support as self-development through online communities of learning. Five other frameworks included references to teachers’ engagement within the academic communities, scholarship of teaching and learning and school cultural activities to support teaching more generally (TC1, TC2, TC3, TC4, TC5). Each of these frameworks tended to position the connection between immediate colleagues and institutional networks as key sources for teachers.

Teachers’ competence to connect with colleagues for support relating to teaching and learning design ideas has been evidenced in the pre-pandemic literature (Bennett et al, 2017). However, the significance of this competence during the intense shift to ERT was critical due to disruptions to supports usually available during design cycles. This is similarly reinforced by Lee (2021) who notes that a break from systematic design during ERT called for teachers to have both available and needs-based support resources for online delivery. It is important to recognise, however, that in practice teachers faced significant uncertainty about the types of development opportunities they should undertake to be prepared during this time.

#### N6. Supports students to navigate the demands of digital environments through design

Five frameworks outlined teacher capabilities to design digital learning environments that considered student needs, engagement, accessibility, and student support resources (DC1, DC2, DC3, TC4, TC7). Three referred specifically to teachers’ capability to integrate student needs in digital aspects of learning design (DC2, DC3, TC7). Though these considerations are not exclusively related to digital demands of the learning environment, addressing student needs, engagement and accessibility are all examples of how a teacher may support students within the digital learning environment. Two frameworks referred to teachers’ capability to provide digital support resources (DC1) or provide links to external digital support resources (TC4) for students. Three teacher capability frameworks also acknowledged the for teachers to consider student needs in the design of learning environments (TC4) or support students learning through inclusive design practices (TC2, TC5).

While not specifically focused on supporting students in digital environments, forms of student-centred design were captured in all ten frameworks. While the digital might be implied in these frameworks, research on digital learning environment design outlines the context-specific design features of digital learning including supporting students’ digital competence (Beetham & Sharpe, 2019). In digital learning environments, supporting students through effective learning design assumes a generally high level of digital competence as teachers need to be able to navigate the digital technology themselves plus also anticipate the varied ways students may engage with the digital environment and the support they may need. In this way, many teachers experienced a digital intensification of teaching and learning considerations during ERT. As digital higher education develops into the future, the complexity of this teacher digital competence warrants further attention and support.

#### N7. Reflects on and evaluates the integration of digital technologies over time

The need for reflection and evaluation of the integration of digital technologies over time were evidenced in three university frameworks. Two referred to the re-design of practices to support a digitally rich learning experience (DC2, DC3) and one referred to the critical review of digital technologies in learning for currency (TC5). Three teaching capability frameworks included reflecting on and applying improvements to learning and design in general without explicit reference to digital technologies (TC1, TC2, TC3).

The research literature illustrates the ways that teachers engage in ongoing reflection on ERT drawing on available reports, feedback and discussion with colleagues and students (Hrastinski, 2021). The continual re-design and development of learning environments through evaluation is clearly conceptualised in teaching frameworks as well as the literature findings. Unlike pedagogy in general, the pace of technological innovation implies that continual learning and reflection about digital technologies and learning environments is a specific digital competence required of teachers. Only one framework acknowledged the importance of digital learning designs being reflective of current digital developments.

### N8. Assesses the time demand and value of digital technology integration

Understanding the time demand and value of digital technology integration was evidenced to some degree in three digital capability frameworks. One identified teacher digital competency as being able to assess the benefits and strengths of digital technologies (DC2). The second takes a wellness position as competent teachers should be able to recognise digital information overload and to disconnect in such a case (DC1). The third shared a similar position with the inclusion of work-life balance and others’ wellbeing when using digital technologies (DC3).

Teachers enacting online delivery models during the ERT period detailed the material constraints on what they could meaningfully invest in their learning environments. The increased workloads and sudden adoption of digital practices led to many teachers to assess the time that goes into both the development and delivery of digital learning. While this period introduced unexpected complexity to teachers, there are important insights from ERT that could provide frames to assist with rapid, value-based decision-making about the integration of digital technologies in learning and teaching (Galyen et al., 2021). Only three of the ten university frameworks evidenced this competency, highlighting an area for further development related to the need to balance the demands that come with embedding digital education practices. Similarly, management of personal wellbeing and demands has been a feature absent in many other theoretical frames of teacher digital competency (Falloon, 2020).

## Discussion

The broad aim of this study was to characterise university teachers’ digital competence from the ERT period and analysed how they are reflected in existing operationalisations of digital competence in university contexts as expressed in institutional frameworks. To do this we developed a conceptual frame from a synthesis of 15 empirical articles that captured teachers' ERT digital competencies. We then applied the conceptual frame as an analytic tool to examine 10 university teaching and digital capability frameworks.

The mass shift to ERT coincided with a progressive shift in higher education to embed digital or technology enhanced learning as standard practice. Thus, understanding the bottom-up change of teachers’ practice is of continued importance to the sector in providing opportunities for reflection and meaningful development of existing policy, resources and supports. From the review of the ERT literature and document analysis of 10 university frameworks, the findings illustrated variations in alignment between the normative statements of ERT digital competence and existing university frameworks. Similarities and differences point to opportunities for universities to build on lessons learnt to support teachers continued development.

Our examination of ten university frameworks found that one normative statement of digital competence, ‘N1 Selects digital technologies that are appropriate for teaching and learning contexts’, was explicitly reflected in all the university frameworks reviewed. This alignment is not surprising given that the selection of appropriate tools is fundamental to all teaching that integrates the use of digital technologies. The nuances of teachers’ selections (based on familiarity, availability, and preferences) that supported their immediate need to stay connected with students draws attention to the social dimension of digital competence. These findings reflect a conceptualisation of digital competence as a contextual social practice, connected to teachers’ experiences, available resources and, more broadly, the culture of the teaching and learning environment (Beckman et al, 2019; Pettersson, 2018).

The remaining normative statements of teachers’ ERT digital competence were found to align with the university frameworks in varied ways. In general, this alignment tended to be implied or partial in broad statements about teaching and learning competence or more specific in statements about digital competence (see N2, N3, N5, N6 & N7). For example, ‘N3 Transfers digital competencies to adapt to a range of teaching contexts and modes’ was evidenced in six university frameworks that broadly described the transfer of pedagogical skill and knowledge across contexts, with only one framework explicitly discussing the transfer of digital competence. By contrast, ‘N2 Accommodates technical challenges in teaching practice and learning designs’ was evidenced in all three digital capability frameworks and none of the teaching capability frameworks. Beyond a technical focus on troubleshooting, this normative statement captured the intersection of teachers’ technical and pedagogical competence through the development of intentional learning designs to minimise technical issues for themselves and their students. For teachers designing and delivering ERT this competence was developed iteratively over time, signalling the growth of their digital competence from solely technical to a combination of technical, contextual, and pedagogical.

The dualism evident in university frameworks reflects contrasting conceptualisations of digital literacy(ies) and competence as either technically or contextually orientated (Kanjanapongpaisal & Antee, 2021; Pangrazio et al, 2020). The bottom-up analysis of ERT teaching practice suggests that developing teachers’ digital competence is technical, contextual, and iterative (Bartolic et al, 2021; Dalipi et al., 2022; Gao & Zhang, 2020; Scherer et al., 2021). Frameworks designed to support the development of teachers’ digital competence should make explicit digital competencies within the broader context(s) of teaching and learning. Such an approach can make explicit the digital and pedagogical possibilities in online, blended and face-to-face contexts to ensure support for teachers across a continuum of technical and pedagogical experiences.

Two normative statements of ERT digital competence illustrated some new aspects when compared with university frameworks. As the research shows, the transition to ERT was not a straightforward experience. For many teachers, digital competence was enacted and developed during an intense period characterised by increased workloads, uncertainty, and anxieties (Watermeyer et al., 2021). Specifically, the ERT literature accounted for the iterative development of teachers’ digital competence in the context of their discipline and the unfolding pandemic. For example, ‘N4 Understands pedagogical demands of discipline, context and digital learning environments’ was partially evidenced in the teaching capability frameworks in terms of pedagogy, discipline and context broadly without reference to the digital. The ERT literature demonstrates that teachers were initially unprepared for negotiating discipline and digital learning environment demands, however they were able to develop their competence over time through experience within communities of practice (Müller et al., 2021; Oliveira et al., 2021; Watermeyer et al., 2021). Research has shown that the teachers who were best prepared to pivot to ERT had prior experience with teaching online (Kaqinari et al., 2022; Scherer et al., 2021). This highlights the importance of understanding the demands of digital learning environments as a key competence. Making the demands of digital learning environments including online and blended modes of teaching and learning within university frameworks explicit could better support teachers and learners.

Finally, ‘N8 Assesses the time demand and value of digital technology integration’ describes the socio-emotional and material constraints that shaped teachers’ digital learning design practices across ERT. The lessons derived from the ERT literature illustrate the situated complexity of teachers’ digital competence (e.g. McQuirter, 2020). While the university frameworks captured notions of wellbeing and digital disconnection as personal competence, such a focus was present in relation to teaching and learning competence. While academics have discussed the increased time demand and workload required to facilitate digital learning designs (Philipsen et al., 2019), this consideration is not adequately reflected in conceptualisations of digital competency frameworks (Falloon, 2020). Being able to assess the relative advantage of a particular digital tool or approach in light of the time investment is critical in making considered design choices. The assessment of time requirements and relative advantage balanced with effects on workload and wellbeing could better frame future decision making as teachers redesign blended or online components of delivery.

In the future, there is opportunity to further develop operationalisations of ERT digital competence in university settings to account for the lessons learnt from the bottom-up change of teaching practice and development of teachers’ digital competencies. The findings of this study illustrate that teachers’ digital competence development brought together technical and pedagogical competence in an iterative and connected design practice. This development was contextual and required significant investment of teachers’ time. As more empirical research on ERT emerges ERT digital competence can be further developed. There will continue to be the need for ERT. Universities’ teaching and digital capability frameworks captured these dynamics in varied ways, suggesting ways that these frameworks might also be updated over time. There is opportunity draw together the technical and pedagogical to support a wider range of digital possibilities including the time and digital demands associated with working across different modes of teaching beyond ERT.

In considering the study’s findings it is important to acknowledge the limitations. We acknowledge that capturing the empirical findings of teachers’ digital competencies across the ERT period is limited because of the time required to engage in and publish research (Lin & Johnson, 2021). Thus, the scope of the literature review likely omitted different contextual arrangements and practices that may come to light in future publications or may simply never be captured. The university frameworks included capture a sample of those publicly available on university websites. While these documents provide a snapshot of practical operationalisations, it is likely that there are other resources available only on university intranet sites that provide a fuller picture.

## Conclusion

The framework we have devised from the available literature helps to understand how university teachers adaptively responded to the new demands wrought by the pandemic. Working under significant pressure, in uncertain times, teachers made decisions about what was feasible and appropriate for themselves and their students so that education provision could continue, albeit in a different form. The framework is a starting point to ensure that teachers are better prepared for ERT in the future. Our analysis of pre-pandemic teaching and digital competency frameworks also identifies some ways in which practical operationalisations might be revised to better connect technical with pedagogical knowledge, make explicit digital possibilities across modes of delivery, and acknowledge the need to protect the wellbeing of university teachers.

## Appendix

See Table 3.

**Table 3:** Literature review (key findings)

Key findings from the literature review	Literature reviewed														
	Damşa et al. (2021)	Mishra et al. (2020)	Oliveira et al. (2021)	Watermeyer et al. (2021)	Bartolic et al. (2021)	Gao and Zhang (2020)	Scull et al. (2020)	Müller et al. (2021)	Väljataga et al. (2020)	Scherer et al. (2021)	Dalipi et al. (2022)	Kaqinari et al. (2022)	Moustakas and Robrade (2022)	Myyry et al. (2022)	Shrestha et al. (2022)
Teachers’ use of technologies was focused on familiar tools	x		x		x	x		x			x	x			
Teachers’ selection of technologies was guided by university technology infrastructures	x		x	x	x	x			x	x	x		x		
Teachers prioritised digital technologies which supported their communication/ connection with students		x				x	x		x				x		x
Teachers’ preparedness for online delivery during covid was varied which impacted the supports utilised	x		x	x	x	x		x		x		x		x	
Teachers relied on many informal practices that were reproduced into their teaching within digital learning environments	x								x		x		x		
Teachers embraced an emergency response delivery rather than completely adopt an online delivery model				x	x			x	x		x			x	
Teachers had to assemble/alter digital learning environments to support their intended pedagogical approaches			x	x		x	x	x		x	x	x	x	x	x
Teachers relied on institutional and collegial networks to refine their digital learning environments	x	x	x	x	x			x	x	x	x	x	x		x
Teachers led student engagement in digital learning environments through pastoral and supportive roles in their teaching				x			x		x				x		
Teachers considered the student experience when engaging with digital learning environments		x			x	x	x	x			x				
Teachers sought to iteratively develop their digital learning environment through reflection and student evaluation of the learning experience	x						x	x			x	x			x
Teachers recognised ERT was an emotional time, with workload and goodwill stretched, impacting the provision of digital learning environments and delivery modes			x	x				x	x	x	x	x	x		x

## References

[CR2] Bartolic SK, Boud D, Agapito J, Verpoorten D, Williams S, Lutze-Mann L, Matzat U, Moreno MM, Polly P, Tai J, Marsh HL, Lin L, Burgess J-L, Habtu S, Rodrigo MMM, Roth M, Heap T, Guppy N (2021). A multi-institutional assessment of changes in higher education teaching and learning in the face of COVID-19. Educational Review.

[CR3] Beckman K, Bennett S, Lockyer L (2019). Reproduction and transformation of students’ technology practice: The tale of two distinctive secondary student cases. British Journal of Educational Technology.

[CR4] Beetham H, Sharpe R (2019). Rethinking pedagogy for a digital age: Principles and practices of design.

[CR5] Bennett S, Agostinho S, Lockyer L (2017). The process of designing for learning: understanding university teachers’ design work. Educational Technology Research and Development.

[CR6] Braun V, Clarke V (2013). Successful qualitative research: A practical guide for beginners.

[CR9] Crawford J, Butler-Henderson K, Rudolph J, Malkawi B, Glowatz M, Burton R, Magni PA, Lam S (2020). COVID-19: 20 countries’ higher education intra-period digital pedagogy responses. Journal of Applied Learning & Teaching.

[CR10] Crompton H (2017). ISTE standards for educators: A guide for teachers and other professionals.

[CR11] Dalipi F, Jokela P, Kastrati Z, Kurti A, Elm P (2022). Going digital as a result of COVID-19: Insights from students’ and teachers’ impressions in a Swedish University. International Journal of Educational Research Open.

[CR12] Damşa C, Langford M, Uehara D, Scherer R (2021). Teachers’ agency and online education in times of crisis. Computers in Human Behavior.

[CR46] Esteve-Mon, F. M., Llopis-Nebot, M. A., & Adell-Segura, J. (2020). Digital teaching competence of university teachers: A systematic review of the literature. *IEEE Revista Iberoamericana de Tecnologias del Aprendizaje, 15*(4), 399–406. 10.1109/RITA.2020.3033225

[CR13] Falloon G (2020). From digital literacy to digital competence: The teacher digital competency (TDC) framework. Educational Technology Research and Development.

[CR14] Galyen K, Meekins D, Kilgore W (2021). Supporting teachers designing in liminality: Embracing a new and flexible way forward. Educational Technology Research and Development.

[CR15] Gao LX, Zhang LJ (2020). Teacher learning in difficult times: examining foreign language teachers’ cognitions about online teaching to tide over COVID-19. Frontiers in Psychology.

[CR16] Hrastinski S (2021). Informing designs for learning when shifting to digital. Educational Technology Research and Development.

[CR17] Janssen J, Stoyanov S, Ferrari A, Punie Y, Pannekeet K, Sloep P (2013). Experts’ views on digital competence: commonalities and differences. Computers & Education.

[CR18] JISC (2019). *Six elements of digital capabilities—Teacher profile (higher education)*. Retrieved from https://repository.jisc.ac.uk/7283/1/BDCP-HET-Profile-110319.pdf

[CR19] Kanjanapongpaisal PG, Antee A (2021). University teachers’ design work: implications for an urgent shift to digital. Educational Technology Research and Development.

[CR20] Kaqinari T, Makarova E, Audran J, Döring AK, Göbel K, Kern D (2022). A latent class analysis of university lecturers’ switch to online teaching during the First COVID-19 lockdown: The role of educational technology, self-efficacy, and institutional support. Education Sciences.

[CR21] Kebritchi M, Lipschuetz A, Santiague L (2017). Issues and challenges for teaching successful online courses in higher education: A literature review. Journal of Educational Technology Systems.

[CR22] Lee D (2021). The process of designing for [Online] learning: Response to Bennett. Educational Technology Research and Development.

[CR23] Lin L, Johnson T (2021). Shifting to digital: informing the rapid development, deployment, and future of teaching and learning: Introduction. Educational Technology Research and Development.

[CR24] McQuirter R (2020). Lessons on change: shifting to online learning during COVID-19. Brock Education Journal.

[CR25] Mishra L, Gupta T, Shree A (2020). Online teaching-learning in higher education during lockdown period of COVID-19 pandemic. International Journal of Educational Research Open.

[CR26] Moustakas L, Robrade D (2022). The challenges and realities of E-learning during COVID-19: The case of University Sport and Physical Education. Challenges.

[CR27] Müller AM, Goh C, Lim LZ, Gao X (2021). COVID-19 emergency elearning and beyond: Experiences and Perspectives of University educators. Education Sciences.

[CR28] Myyry L, Kallunki V, Katajavuori N, Repo S, Tuononen T, Anttila H, Kinnunen P, Haarala-Muhonen A, Pyörälä E (2022). COVID-19 accelerating academic teachers’ digital competence in distance teaching. Frontiers in Education.

[CR29] Oliveira G, Grenha Teixeira J, Torres A, Morais C (2021). An exploratory study on the emergency remote education experience of higher education students and teachers during the COVID-19 pandemic. British Journal of Educational Technology.

[CR30] Pangrazio L, Godhe A-L, Ledesma AGL (2020). What is digital literacy? A comparative review of publications across three language contexts. E-Learning and Digital Media.

[CR31] Pettersson F (2018). On the issues of digital competence in educational contexts—A review of literature. Education and Information Technologies.

[CR32] Philipsen B, Tondeur J, Pareja Roblin N, Vanslambrouck S, Zhu C (2019). Improving teacher professional development for online and blended learning: A systematic meta-aggregative review. Educational Technology Research and Development.

[CR33] Press N, Arumugam PP, Ashford-Rowe K (2019). Defining digital literacy: A Case Study of Australian Universities.

[CR34] Redecker C (2017). European framework for the digital competence of educators: DigCompEdu. Publications Office.

[CR35] Scherer R, Howard SK, Tondeur J, Siddiq F (2021). Profiling teachers’ readiness for online teaching and learning in higher education: Who’s ready?. Computers in Human Behavior.

[CR36] Scull J, Phillips M, Sharma U, Garnier K (2020). Innovations in teacher education at the time of COVID19: An Australian perspective. Journal of Education for Teaching.

[CR37] Selwyn N (2007). The use of computer technology in university teaching and learning: A critical perspective: a critical look at computer use in higher education. Journal of Computer Assisted Learning.

[CR38] Sharpe R, Bennett S, Varga-Atkins T, Sharpe R, Bennett S, Varga-Atkins T (2022). Introduction to the handbook of digital higher education. Handbook of digital higher education.

[CR39] Shrestha S, Haque S, Dawadi S, Giri RA (2022). Preparations for and practices of online education during the Covid-19 pandemic: A Study of Bangladesh and Nepal. Education and Information Technologies.

[CR40] Spante M, Hashemi SS, Lundin M, Algers A (2018). Digital competence and digital literacy in higher education research: Systematic review of concept use. Cogent Education.

[CR41] Tondeur J, van Braak J, Sang G, Voogt J, Fisser P, Ottenbreit-Leftwich A (2012). Preparing pre-service teachers to integrate technology in education: A synthesis of qualitative evidence. Computers & Education.

[CR42] Väljataga T, Poom-Valickis K, Rumma K, Aus K (2020). Transforming higher education learning ecosystem: Teachers’ perspective. Interaction Design and Architecture(s).

[CR43] Watermeyer R, Crick T, Knight C, Goodall J (2021). COVID-19 and digital disruption in UK Universities: Afflictions and affordances of emergency online migration. Higher Education.

[CR44] Webb A, McQuaid RW, Webster CWR (2021). Moving learning online and the COVID-19 Pandemic: A University response. World Journal of Science, Technology and Sustainable Development.

[CR45] Zhao Y, Pinto Llorente AM, Sánchez Gómez MC (2021). Digital competence in higher education research: A systematic literature review. Computers & Education.

